# Plasminogen activator inhibitor-1 deficiency enhances subchondral osteopenia after induction of osteoarthritis in mice

**DOI:** 10.1186/s12891-017-1752-5

**Published:** 2017-09-11

**Authors:** Akihiro Moritake, Naoyuki Kawao, Kiyotaka Okada, Kohei Tatsumi, Masayoshi Ishida, Katsumi Okumoto, Osamu Matsuo, Masao Akagi, Hiroshi Kaji

**Affiliations:** 1 Department of Orthopaedic Surgery, Kindai University Faculty of Medicine, Osakasayama, Japan; 2 Department of Physiology and Regenerative Medicine, Kindai University Faculty of Medicine, 377-2 Ohnohigashi, Osakasayama, Osaka, 589-8511 Japan; 3 Life Science Research Institute, Kindai University, Osakasayama, Japan

**Keywords:** Osteoarthritis, Osteoclast, Ovariectomy, Plasminogen activator inhibitor-1, Subchondral bone

## Abstract

**Background:**

Subchondral osteopenia is important for the pathophysiology of osteoarthritis (OA). Although previous studies suggest that plasminogen activator inhibitor-1 (PAI-1), an inhibitor of fibrinolysis, is related to bone metabolism, its role in OA remains unknown. We therefore investigated the roles of PAI-1 in the subchondral bone in OA model mice.

**Methods:**

Wild type (WT) and PAI-1-deficient (KO) mice were ovariectomized (OVX), and then destabilization of the medial meniscus (DMM) surgery was performed.

**Results:**

DMM and OVX significantly decreased the trabecular bone mineral density of the subchondral bone evaluated by quantitative computed tomography in PAI-1 KO mice. The effects of OVX and/or PAI-1 deficiency on the OARSI score for the evaluation of the progression of knee degeneration were not significant. PAI-1 deficiency significantly augmented receptor activator nuclear factor κB ligand mRNA levels enhanced by IL-1β in mouse primary osteoblasts, although it did not affect osteoblast differentiation. Moreover, PAI-1 deficiency significantly increased osteoclast formation from mouse bone marrow cells.

**Conclusion:**

We showed that PAI-1 deficiency accelerates the subchondral osteopenia after induction of OA in mice. PAI-1 might suppress an enhancement of bone resorption and subsequent subchondral osteopenia after induction of OA in mice.

## Background

Osteoarthritis (OA) is one of the most frequent diseases in orthopedic clinics. Despite being a disease with a major impact on the medical economy, OA has been generally recognized as a disease that occurs with aging, and its treatment has been only limited to artificial joint replacement when the disease progresses to a severe stage. Although OA has been mainly considered to be due to the wear of cartilage from mechanical stress [1], the prevalence of OA has been reported to be increased in postmenopausal women [2–4]. Moreover, a previous study suggested that osteoporosis aggravates cartilage damage in knee OA model rats [5]. These findings suggest the possibility that bone metabolism and osteoporosis affects the progression of arthropathy.

Changes in bone around the joint, especially the subchondral bone, have recently been suggested to be involved in the disease condition from a relatively early stage in OA. Particularly, the subchondral bone is influenced by a high bone turnover state. Radin et al. suggested that the integrity of the articular cartilage depends on the underlying bone strength [6]. They speculated that cartilage degeneration is prone to progress by load stress in the osteoporotic state of the subchondral bone. Accelerated bone turnover by osteoblasts and osteoclasts is indispensable for remodeling of the subchondral bone in OA [7]. Moreover, a recent study indicated that the promotion of subchondral bone turnover is accompanied by specific structural changes in the subchondral trabecular bone of the OA joint [8]. These findings suggest that maintenance of bone mass in the subchondral bone may be important for the treatment of arthropathy [9, 10]. However, the details of the mechanisms of subchondral osteopenia after induction of OA and the role of estrogen deficiency remain unclear.

Plasminogen activator inhibitor-1 (PAI-1) is a serine protease inhibitor that primarily inhibits tissue- and urokinase-type plasminogen activators, and acts as an inhibitor of fibrinolysis [11, 12]. PAI-1 possesses a fibrinolysis-independent function in various tissues [12]. PAI-1 is expressed in various cells, including bone and cartilage, as well as in the extracellular matrix. We previously revealed that PAI-1 is involved in osteopenia induced by diabetes and glucocorticoid excess as well as diabetes-induced delayed bone repair using PAI-1-deficient mice [13–15]. However, the roles of PAI-1 in the pathogenesis of OA and osteoclastic bone resorption still remain unknown.

In the present study, we therefore investigated the roles of PAI-1 in the subchondral bone and cartilage changes in OA model mice using wild-type (WT) and PAI-1-deficient (KO) mice.

## Methods

### Materials

Macrophage colony-stimulating factor (M-CSF) and receptor activator of nuclear factor-κB ligand (RANKL) were purchased from Wako (Osaka, Japan). SB431542, interleukin-1β (IL-1β) and transforming growth factor-β_1_ (TGF-β_1_) were obtained from Tocris (Bristol, UK), R&D systems (Minneapolis, MN, USA) and Sigma (St Louis, MO, USA), respectively. Bone morphogenetic protein-2 (BMP-2) was obtained from Pfizer Inc. (Groton, CT, USA).

### Animals

PAI-1 KO and counterpart WT mice were used in this study. WT and PAI-1 KO mice were kindly provided by D. Collen (University of Leuven, Leuven, Belgium) and were bred at Kindai University Faculty of Medicine animal facility. The genetic background of all mice was mixed C57BL/6 J (81.25%) and 129/SvJ (18.75%). Female WT and PAI-1 KO mice were randomly divided into four groups: WT/Sham (*n* = 11), WT/ovariectomy (OVX) (n = 11), PAI-1 KO/Sham (*n* = 10) and PAI-1 KO/OVX (n = 11). Seven-week-old female WT and PAI-1 KO mice were ovariectomized or sham-operated as previously described [16]. One week after the OVX or sham surgery, all mice received destabilization of the medial meniscus (DMM) surgery on the right knee due to induction of OA and sham surgery on the left knee. Eight weeks after the DMM surgery, these mice were subjected to analysis. Food and water were provided ad libitum. The room temperature was kept at 24 ± 1 °C with a 12 h:12 h light/dark cycle. All experiments were performed according to the guidelines of the National Institutes of Health and the institutional rules for the use and care of laboratory animals at Kindai University.

### DMM surgery

The DMM model, which provided high reproducibility and a slower progression of OA in mice, was used for evaluation of changes in subchondral bone and cartilage in the OA state in WT and PAI-1 KO mice. DMM surgery was performed on the right knee of mice as previously described [17]. Briefly, under anesthesia induced by 2% isoflurane, the anterior skin was cut by 5 mm to expose the joint capsule in mice. To expose the medial meniscus, the joint capsule immediately medial to the patellar tendon was incised. After transection of the meniscotibial ligament, skin was closed sterilely. A sham surgery was performed on the left knee using the same procedure with no transection of the meniscotibial ligament.

### Quantitative computed tomography (qCT) analysis

For qCT analysis of the bone mineral density (BMD), mice were anesthetized using 2% isoflurane and scanned using an experimental animal CT system (LaTheta LCT-200, Hitachi Aloka Medical, Tokyo, Japan) [18]. CT scans were performed at a tube voltage of 50 kVp, a tube current of 500 μA, an integration time of 3.6 ms, an axial field of view of 48 mm, and an isotopic voxel size of 24 μm. To assess the trabecular BMD of the tibial subchondral bone, regions of interest (ROI) were defined as 240-μm (10-slice) segments from 72 μm proximal to the end of the proximal growth plate towards the joint. This ROI was 200 μm distal towards the joint, compared to ROI for the assessment of the trabecular BMD for systemic osteopenia in tibia. An increase in bone mineral content occurs in the most proximate subchondral region of the cartilage tissues in the knee. We therefore employed the most proximate trabecular bone region with a decrease in BMD with DMM and OVX in our preliminary experiments as the ROI for the measurement of BMD of the subchondral bone. Bone parameters were analyzed using LaTheta software (version3.40). A threshold density of 160 mg/cm^3^ was set to distinguish mineralized from unmineralized tissue. The density range was calibrated daily with a phantom supplied by the manufacturer.

### Histology analysis

Mice were euthanized with excess isoflurane 8 weeks after DMM surgery. The knee samples were removed, fixed in 4% paraformaldehyde for 24 h, and decalcified in 22.5% formic acid and 340 mM sodium citrate solution for 24 h. After demineralization, specimens were embedded in paraffin and cut into 5-μm sections. The sections were stained with hematoxylin/eosin and photographed under a microscope (BZ-9000, Keyence, Osaka, Japan). The sections were stained with Safranin-O and fast green, and subsequently counterstained with hematoxylin. Each knee was evaluated for the progression of arthropathy with 6 grades using the Osteoarthritis Research Society International (OARSI) histological scoring system [19].

### Quantitative real-time PCR

Total RNA was extracted from primary osteoblasts using the RNeasy Mini Kit (Qiagen, Tokyo, Japan). One μg total RNA was reverse transcribed using a High-Capacity cDNA Reverse Transcription Kit (Applied Biosystems, Foster, CA, USA). Quantitative real-time PCR was performed using StepOnePlus and the Fast SYBR Green PCR Master Mix (Life Technologies, Tokyo, Japan) as previously described [13]. A list of primers used is shown in Table 1. The specific mRNA amplification of the target was determined as the Ct value, which was followed by normalization by the glyceraldehyde 3-phosphate dehydrogenase (GAPDH) mRNA level.

**Table 1:** Primers used for real-time PCR experiments

Gene		Primer sequence
PAI-1	Forward	5′-TTCAGCCCTTGCTTGCCTC-3’
	Reverse	5′-ACACTTTTACTCCGAAGTCGGT-3’
TGF-β_1_	Forward	5′-CCTCTGTCACCTGCTCAACA-3’
	Reverse	5′-GATGAATTGGCGTGGAATCT-3’
Runx2	Forward	5′-AAATGCCTCCGCTGTTATGAA-3’
	Reverse	5′-GCTCCGGCCCACAAATCT-3’
Osterix	Forward	5′-AGCGACCACTTGAGCAAACAT-3’
	Reverse	5′-GCGGCTGATTGGCTTCTTCT-3’
ALP	Forward	5′-ATCTTTGGTCTGGCTCCCATG-3’
	Reverse	5′-TTTCCCGTTCACCGTCCAC-3’
OCN	Forward	5′-CCTGAGTCTGACAAAGCCTTCA-3’
	Reverse	5′-GCCGGAGTCTGTTCACTACCTT-3’
RANKL	Forward	5′- CACAGCGCTTCTCAGGAGCT-3’
	Reverse	5′-CATCCAACCATGAGCCTTCC-3’
OPG	Forward	5′-AGTCCGTGAAGCAGGAGT-3’
	Reverse	5′-CCATCTGGACATTTTTTGCAAA-3’
GAPDH	Forward	5′-AGGTCGGTGTGAACGGATTTG-3’
	Reverse	5′-GGGGTCGTTGATGGCAACA-3’

*PAI-1* plasminogen activator inhibitor-1, *TGF-β*_*1*_ transforming growth factor-β1, *ALP* alkaline phosphatase, *OCN* osteocalcin *RANKL* receptor activator of nuclear factor-κB ligand, *OPG* osteoprotegerin, *GAPDH* glyceraldehyde-3-phosphate dehydrogenase

### Primary osteoblasts and bone marrow stromal cells

Primary osteoblastic cells were obtained from the calvaria of 3-day-old female WT and PAI-1 KO mice, as previously described [20]. The calvaria was removed from soft tissue, and digested 4 times with 1 mg/mL collagenase and 0.25% trypsin for 20 min at 37 °C. Cells from second, third and fourth digestions were collected and grown in Minimum Essential Medium Alpha Modification (α-MEM; Wako) with 10% fetal bovine serum (FBS; HyClone, Logan, UT, USA). The medium was changed twice a week.

Bone marrow stromal cells were obtained from female WT and PAI-1 KO mice as previously described [20]. Briefly, femur and tibia were removed and subsequently the bone marrow cells were flushed out into Dulbecco’s Modified Eagle’s Medium (DMEM; Wako) with 10% FBS. The nonadherent cells were removed by washing using phosphate-buffered saline.

### Osteoclast formation

Bone marrow cells were collected from the femur and tibia of 8-week-old female WT and PAI-1 KO mice. Analysis of osteoclast formation was performed as previously reported [21]. Briefly, bone marrow cells were plated in a 24-well plate (5.0 × 10^5^ cells/well) and cultured in α-MEM with 10% FBS and 50 ng/mL M-CSF for 3 days at 37 °C. Then, osteoclasts were formed in α-MEM with 10% FBS, 50 ng/mL M-CSF and 50 ng/mL RANKL for a further 3 days at 37 °C. Detection of osteoclasts was performed using a tartrate-resistant acid phosphatase (TRAP) staining kit (Wako). The numbers of TRAP-positive multinucleated cells (MNCs) were counted in a certain area within the well.

### Statistical analysis

All data were expressed as the mean ± SEM. Two-way ANOVAs were used to compare the effects of OVX and DMM on both mouse genotypes (WT and PAI-1 KO). When significant differences were observed, individual means were compared using Tukey-Kramer post hoc tests. Statistical values at *P* < 0.05 were considered to be significant. All statistical analyses were performed using the Prism software, version 7 (GraphPad Software, Inc., La Jolla, CA, USA).

## Results

### Effects of DMM, OVX and PAI-1 deficiency on BMD of subchondral bone in mice

**Fig. 1:**
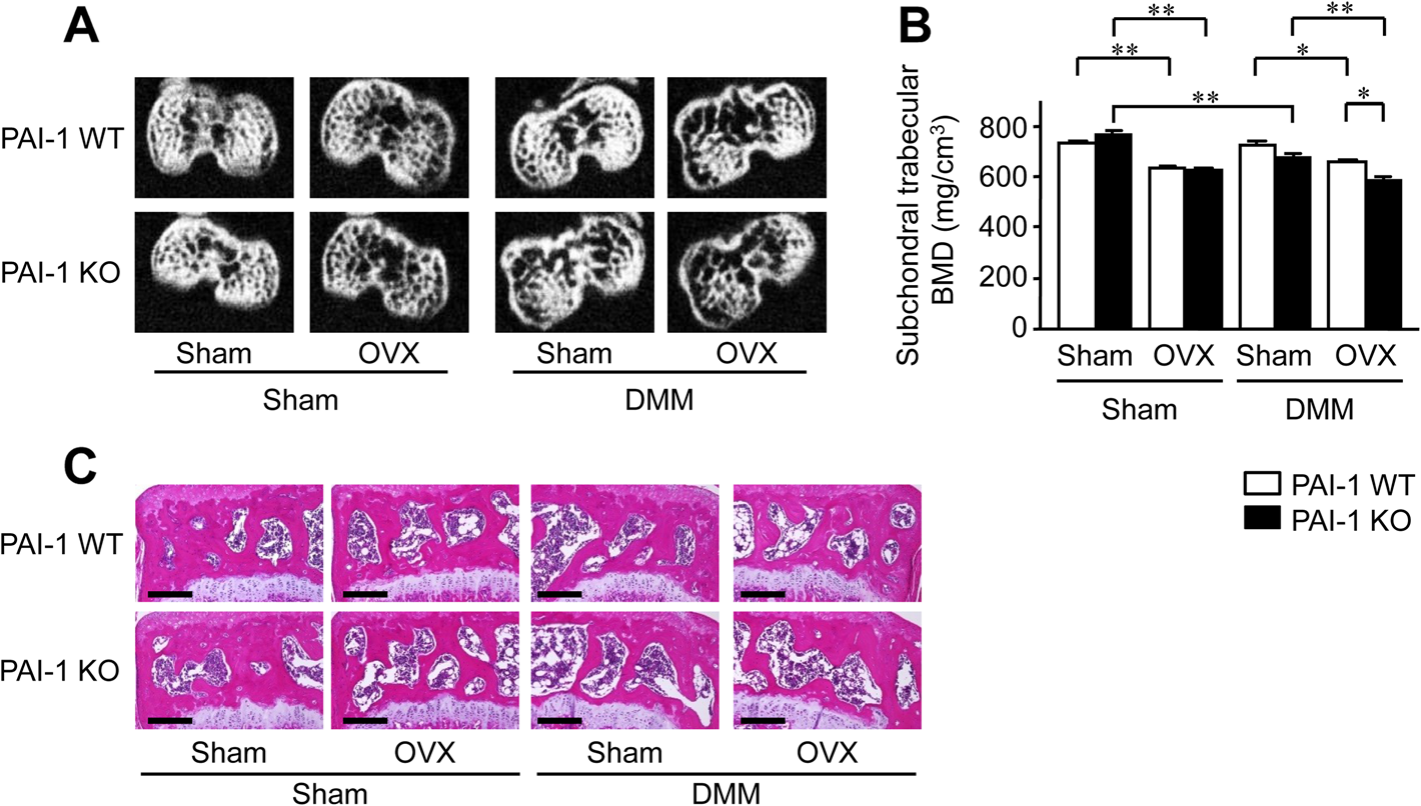
Effects of DMM, OVX and PAI-1 deficiency on the BMD of the tibial subchondral bone in mice. **a** qCT images of the subchondral bone in WT and PAI-1 KO mice 8 weeks after DMM or sham surgery with or without OVX. **b** The trabecular BMD of the subchondral bone was assessed by qCT in WT and PAI-1 KO mice 8 weeks after DMM or sham surgery with or without OVX. Regions of interest were defined as 240-μm (10-slice) segments from 72 μm proximal to the end of the proximal growth plate towards the joint. Data represent the mean ± SEM of 10–11 mice. **p < 0.01 and *p < 0.05. **c** Hematoxylin and Eosin-stained sections from the subchondral bone in WT and PAI-1 KO mice 8 weeks after DMM or sham surgery with or without OVX. Scale bars = 200 μm

We evaluated the trabecular BMD of the tibial subchondral bone, in which ROI were defined as 240 μm (10-slice) segments from 72 μm proximal to the end of the proximal growth plate towards the joint using quantitative CT analysis. OVX significantly decreased the trabecular BMD of the subchondral bone in WT and PAI-1 KO mice (Fig. 1a, b). DMM significantly decreased the trabecular BMD of the subchondral bone in PAI-1 KO mice, although DMM did not affect it in WT mice with or without OVX. PAI-1 deficiency significantly augmented a decrease in the trabecular BMD of the subchondral bone in mice with OVX and DMM (Fig. 1a, b). Histological analysis findings were compatible with those results in qCT (Fig. 1c).

### Effects of DMM, OVX and PAI-1 deficiency on articular cartilage degeneration in mice

**Fig. 2:**
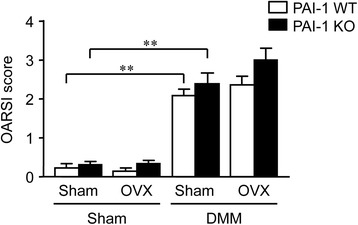
Effects of DMM, OVX and PAI-1 deficiency on articular cartilage degeneration in mice. Cartilage degeneration was assessed using the OARSI histological scoring system in WT and PAI-1 KO mice 8 weeks after DMM or sham surgery with or without OVX. Each knee was evaluated using 6 grades for the progression of arthropathy. Data represent the mean ± SEM of 10–11 mice. **p < 0.01

Next, we examined the effects of OVX, DMM and PAI-1 deficiency on articular cartilage degeneration in mice. The progression of arthropathy at the knees was evaluated by an OARSI scoring system, which is a widely used method for the evaluation of osteoarthritis. As shown in Fig. 2, there were no significant differences in the OARSI score between WT and PAI-1 KO mice with or without OVX. DMM significantly increased the OARSI score in WT and PAI-1 KO mice, although OVX did not affect the OARSI score. OARSI scores seemed to be elevated in PAI-1 KO mice, compared to those in WT mice, especially in mice with both OVX and DMM, although their differences were not statistically significant.

### Roles of IL-1β and PAI-1 deficiency in primary osteoblasts

**Fig. 3:**
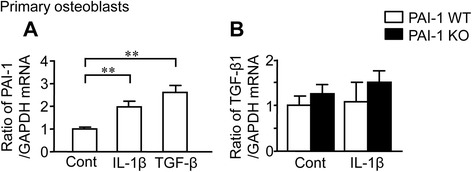
Effects of IL-1β on the expressions of PAI-1 and TGF-β_1_ in primary mouse osteoblasts. **a** Primary osteoblasts were obtained from 3-day-old female WT mice. Total RNA was extracted from primary osteoblasts cultured with or without 10 ng/mL IL-1β or 1 ng/ml TGF-β_1_ for 24 h. The levels of PAI-1 or GAPDH mRNA were assessed by real-time PCR. Data represent the mean ± SEM. *n* = 6 for each group. **p < 0.01. **b** Primary osteoblasts were obtained from 3-day-old female WT and PAI-1 KO mice. Total RNA was extracted from primary osteoblasts cultured with or without 10 ng/mL IL-1β for 24 h. Then, the levels of TGF-β_1_ or GAPDH mRNA were assessed by real-time PCR. Data represent the mean ± SEM. *n* = 6 for each group

**Fig. 4:**
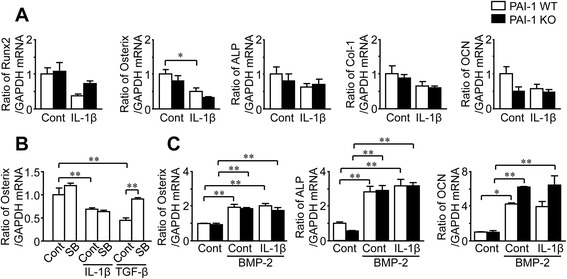
Effects of PAI-1 deficiency on osteoblast differentiation in mouse osteoblasts and bone marrow cells. **a** Primary osteoblasts were obtained from 3-day-old female WT and PAI-1 KO mice. Total RNA was extracted from primary osteoblasts cultured with or without 10 ng/mL IL-1β or 1 ng/ml TGF-β_1_ for 24 h. The levels of Runx2, Osterix, ALP, Col-1, OCN or GAPDH mRNA were assessed by real-time PCR. Data represent the mean ± SEM. n = 6 for each group. *p < 0.05. **b** Primary osteoblasts were obtained from 3-day-old female WT mice. Total RNA was extracted from primary osteoblasts cultured with or without 10 ng/mL IL-1β or 1 ng/ml TGF-β_1_ in the presence or absence of 1 μM SB421542 for 24 h. The levels of Osterix or GAPDH mRNA were assessed by real-time PCR. Data represent the mean ± SEM. n = 6 for each group. **p < 0.01. **c** Bone marrow cells were obtained from 8-week-old female WT and PAI-1 KO mice. Total RNA was extracted from the bone marrow cells cultured with or without 200 ng/ml BMP-2 or 10 ng/mL IL-1β for 48 h. The levels of Osterix, ALP, OCN or GAPDH mRNA were assessed by real-time PCR. Data represent the mean ± SEM. *n* = 5 for each group. **p < 0.01 and *p < 0.05

We investigated the mechanisms by which PAI-1 deficiency augments subchondral osteopenia induced by DMM in mice using mouse primary osteoblasts from WT and PAI-1 KO mice. IL-1β and TGF-β are important and representative factors for the pathogenesis of OA [22]. We therefore examined the roles of IL-1β and TGF-β signals in WT and PAI-1 KO mice to clarify the roles of PAI-1 in the pathological state of OA in vitro. Both IL-1β and TGF-β significantly enhanced PAI-1 mRNA levels in mouse osteoblasts (Fig. 3a). However, IL-1β did not affect TGF-β mRNA levels in these cells from both WT and PAI-1 KO mice (Fig. 3b). Next, we examined the effects of IL-1β and PAI-1 deficiency on osteoblast differentiation in mouse osteoblasts from WT and PAI-1 KO mice. As shown in Fig. 4a, IL-1β significantly decreased Osterix mRNA levels in WT mice, although its effects on other osteogenic genes, such as Runx2, alkaline phosphatase (ALP), type I collagen (Col-1) and osteocalcin (OCN) were not significant. PAI-1 deficiency did not affect Osterix mRNA levels suppressed by IL-1β (Fig. 4a). Moreover, SB431542, an inhibitor of activin-like kinase (ALK)5, TGF-β receptor type I kinase, did not affect Osterix mRNA levels suppressed by IL-1β, although it significantly blunted Osterix mRNA levels suppressed by TGF-β in osteoblasts (Fig. 4b). We next investigated the involvement of PAI-1 in osteoblast differentiation from mouse bone marrow cells in the presence or absence of IL-1β. As shown in Fig. 4c, PAI-1 deficiency did not affect the mRNA levels of Osterix, ALP and OCN in bone marrow cells treated with BMP-2 in the presence or absence of IL-1β, suggesting that PAI-1 deficiency does not affect osteoblastic differentiation from mouse bone marrow cells.

### Effects of PAI-1 deficiency on osteoclast formation in mice

**Fig. 5:**
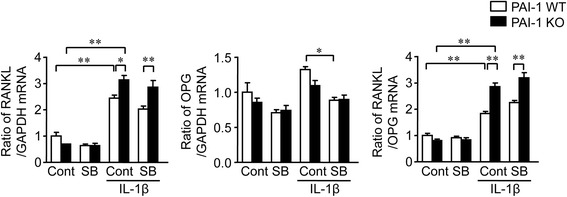
Effects of PAI-1 deficiency on the expression of RANKL and OPG in mouse osteoblasts. Primary osteoblasts were obtained from 3-day-old female WT and PAI-1 KO mice. Total RNA was extracted from primary osteoblasts cultured with or without 10 ng/mL IL-1β or 1 ng/ml TGF-β_1_ in the presence or absence of 1 μM SB421542 for 24 h. The levels of RANKL, OPG or GAPDH mRNA were assessed by real-time PCR. Data represent the mean ± SEM. n = 6 for each group. **p < 0.01 and *p < 0.05

Finally, we investigated the effects of PAI-1 deficiency on osteoclast formation in WT and PAI-1 KO mice. RANKL is a crucial factor for osteoclast formation and activity, and osteoprotegerin (OPG) inhibits osteoclast formation by binding to RANKL. RANKL and OPG are the chief regulators of bone resorption. As shown in Fig. 5, IL-1β significantly elevated RANKL mRNA levels as well as the ratio of RANKL/OPG mRNA in osteoblasts from WT mice. PAI-1 deficiency significantly augmented this IL-1β-enhanced RANKL mRNA level and RANKL/OPG mRNA ratio in osteoblasts. However, SB431542, a TGF-β signaling inhibitor, did not affect the IL-1β-enhanced RANKL mRNA level and RANKL/OPG mRNA ratio in osteoblasts from mice with or without PAI-1 deficiency.

**Fig. 6:**
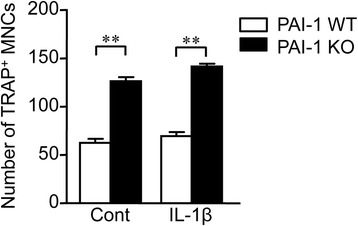
Effects of PAI-1 deficiency on osteoclast formation from mouse bone marrow cells. After bone marrow cells obtained from WT and PAI-1 KO mice cultured with M-CSF (50 ng/ml) for 3 days, cells were treated with or without IL-1β in the presence of M-CSF (50 ng/ml) and RANKL (50 ng/ml) for 3 days. Then, TRAP-positive MNCs were counted. Data represent the mean ± SEM. n = 6 for each group. **p < 0.01

We examined the effects of PAI-1 deficiency of osteoclast formation from mouse bone marrow cells obtained from WT and PAI-1 KO mice. PAI-1 deficiency significantly increased the number of TRAP-positive MNCs in the presence or absence of IL-1β in mouse bone marrow cell cultures in the presence of M-CSF and RANKL (Fig. 6).

## Discussion

In the present study, we showed that PAI-1 deficiency augmented subchondral osteopenia after induction of OA and/or OVX in mice. PAI-1 deficiency enhanced osteoclast formation from mouse bone marrow cells as well as RANKL expression induced by IL-1β in mouse osteoblasts, although it did not affect osteoblast differentiation suppressed by IL-1β.

Subchondral osteopenia as well as the destruction of joint cartilage are important for the pathogenesis of OA [23, 24]. The initial change of cartilage induced by excessive mechanical stress in OA may cause the changes to the subchondral bone through inflammatory cytokines, such as IL-1β [25]. On the other hand, the initial change of the subchondral bone and subsequent local cytokine release might induce cartilage degeneration. The contribution of both mechanisms in the pathogenesis of OA is still controversial. In the present study, DMM significantly enhanced subchondral bone osteopenia in PAI-1-deficient mice with or without OVX, suggesting that knee OA progression induces subchondral osteopenia at least PAI-1 deficient state in mice. We speculate that the trabecular BMD decrease in the subchondral bone represents local subchondral osteopenia after induction of OA in this study. However, further analysis using micro CT with higher resolution will be necessary for the detection of concrete subchondral bone changes in mice.

We and others have previously reported the pathophysiological roles of PAI-1 in bone and cartilage [13–15]. Rundle et al. reported that the fracture callus was enlarged in PAI-1-deficient mice [26]. Moreover, we showed that circulating PAI-1 might be involved in diabetic osteopenia and delayed bone repair as well as glucocorticoid-induced osteoporosis in mice [13–15]. In that study, exogenous addition of PAI-1 suppresses osteoblast differentiation in mouse osteoblasts [13]. In contrast, in the present study, PAI-1 deficiency significantly augmented subchondral osteopenia after induction of OA with or without OVX in mice. These findings suggest that PAI-1 might protect against subchondral osteopenia after induction of OA in mice.

IL-1β plays central roles as an inflammatory cytokine in the pathogenesis of OA [27]. IL-1β induces bone resorption, which is related to subchondral osteopenia in OA. In the present study, PAI-1 deficiency did not affect genes related to osteoblast differentiation that are suppressed by IL-1β in mouse osteoblasts, although IL-1β significantly enhanced the expression of PAI-1. On the other hand, PAI-1 deficiency significantly enhanced osteoclast formation from mouse bone marrow cells in the presence of M-CSF and RANKL. Moreover, PAI-1 deficiency significantly augmented the expression of RANKL and the ratio of RANKL/OPG mRNA in mouse primary osteoblasts. These findings suggest that PAI-1 protects against bone resorption in the subchondral bone after induction of OA, although PAI-1 does not affect osteoblast differentiation suppressed after induction of OA. This finding might partly explain the mechanisms by which PAI-1 deficiency augments subchondral osteopenia in mice. Several studies indicate that increases in osteoclast number and bone resorption are observed at the subchondral bone in patients or animals with OA [23, 24, 28–30]. Moreover, PAI-1 deficiency promoted bone remodeling during fracture healing in mice [26]. Taken together, PAI-1 might suppress the enhancement of bone resorption and subsequent subchondral osteopenia after induction of OA in mice. Subchondral osteopenia enhanced by PAI-1 deficiency seemed to augment cartilage degeneration, although the statistical differences were not significant in the present study.

Previous studies indicate that TGF-β is involved in the pathogenesis of OA [28, 31]. Moreover, TGF-β is related to subchondral osteopenia after induction of OA [28]. In the present study, inhibition of TGF-β signaling by an ALK5 inhibitor did not affect the effects of IL-1β on osteoblast differentiation and RANKL expression in mouse osteoblasts. Moreover, IL-1β did not affect TGF-β expression in osteoblasts from both WT and PAI-1 KO mice. These findings indicate that the effects of PAI-1 deficiency on the enhancement of osteoclastic bone resorption are independent from TGF-β signaling in mice.

Postmenopausal osteoporosis may be a risk factor for the development of knee OA [32]. Several studies suggest that OA is accelerated in postmenopausal women [3, 4]. The influence of OVX on OA progression in animal studies is still controversial [33, 34], although Miyatake et al. reported that OVX exacerbates the severity of OA induced by treadmill exercise in mice [35]. Our data showed that OVX significantly enhances subchondral osteopenia induced by DMM in PAI-1-deficient mice, but not in WT mice. From these findings, we speculate that OVX might enhance subchondral osteopenia when the suppression of PAI-1 on osteoclast formation was cancelled. There were neither additive nor synergistic effects in the influences of OVX and DMM on the subchondral trabecular BMD in the present study. Although the reason remains unknown, IL-1β has been known to be involved in the pathogenesis of osteopenia induced by both estrogen deficiency and OA [27, 36].

Our study suggested that an induction of local endogenous PAI-1 at the subchondral bone suppresses subchondral osteopenia after induction of OA in mice. Clinical evidence indicates that PAI-1 is related to the pathophysiology of various diseases, such as diabetes, cardiovascular diseases, glucocorticoid excess and cancer, as a malignant adipocytokine [12]. In contrast, the protective role of PAI-1 of the subchondral bone in the pathophysiology of knee OA is contrary to the action of PAI-1 as a malignant adipocytokine [12]. Further studies are necessary to clarify the roles of PAI-1 in tissues such as the cartilage and synovial cells to investigate the detailed roles of PAI-1 in the pathogenesis of OA.

## Conclusions

In conclusion, we demonstrated that PAI-1 deficiency enhances subchondral osteopenia after induction of OA in mice. An enhancement of IL-1β-induced RANKL expression and subsequent osteoclast formation by PAI-1 deficiency might be related to this mechanism.

## Abbreviations

ALK: Activin-like kinase
ALP: Alkaline phosphatase
BMD: Bone mineral density
BMP-2: Bone morphogenetic protein-2
Col-1: Type I collagen
DMEM: Dulbecco’s modified eagle’s medium
DMM: Destabilization of the medial meniscus
FBS: Fetal bovine serum
GAPDH: Glyceraldehyde 3-phosphate dehydrogenase
IL-1β: Interleukin-1β
M-CSF: Macrophage colony-stimulating factor
MNCs: Multinucleated cells
OA: Osteoarthritis
OARSI: Osteoarthritis research society international
OCN: Osteocalcin
OPG: Osteoprotegerin
OVX: Ovariectomy
PAI-1 KO: Plasminogen activator inhibitor-1-deficient
PAI-1: Plasminogen activator inhibitor-1
QCT: Quantitative computed tomography
RANKL: Receptor activator of nuclear factor-κB ligand
ROI: Regions of interest
TGF-β_1_: Transforming growth factor-β_1_
TRAP: Tartrate-resistant acid phosphatase
WT: Wild type
α-MEM: Minimum essential medium alpha modification
